# Protocol for image registration of correlative soft X-ray tomography and super-resolution structured illumination microscopy images

**DOI:** 10.1016/j.xpro.2021.100529

**Published:** 2021-05-06

**Authors:** Nina Vyas, Stephan Kunne, Thomas M. Fish, Ian M. Dobbie, Maria Harkiolaki, Perrine Paul-Gilloteaux

**Affiliations:** 1 Beamline B24, Diamond Light Source, Harwell Science and Innovation Campus, Didcot, Oxfordshire, OX11 0DE, UK; 2 Université de Nantes, CHU Nantes, CNRS UMR 6291, INSERM UMR 1087, L'institut du thorax, 44000 Nantes, France; 3 Micron Advanced Imaging Consortium, Department of Biochemistry, University of Oxford, South Parks Road, Oxford OX1 3QU, UK; 4 Université de Nantes, CHU Nantes, Inserm, CNRS, SFR Santé, Inserm UMS 016, CNRS UMS 3556, 44000 Nantes, France

**Keywords:** Microscopy, Structural Biology

## Abstract

Correlation of 3D images acquired on different microscopes can be a daunting prospect even for experienced users. This protocol describes steps for registration of images from soft X-ray absorption contrast imaging and super-resolution fluorescence imaging of hydrated biological materials at cryogenic temperatures. Although it is developed for data generated at synchrotron beamlines that offer the above combination of microscopies, it is applicable to all analogous imaging systems where the same area of a sample is examined using successive non-destructive imaging techniques.

For complete details on the use and execution of this protocol, please refer to Kounatidis et al. (2020).

## Highlights

• This protocol demonstrates how to correlate X-ray and fluorescence microscopy data
• Developed for cryo-soft X-ray tomography and structured illumination microscopy
• Open-source software eC-CLEM is used for correlation
• Information on correcting for the chromatic shift and stage shift is also provided

## Graphical abstract

**Figure:**
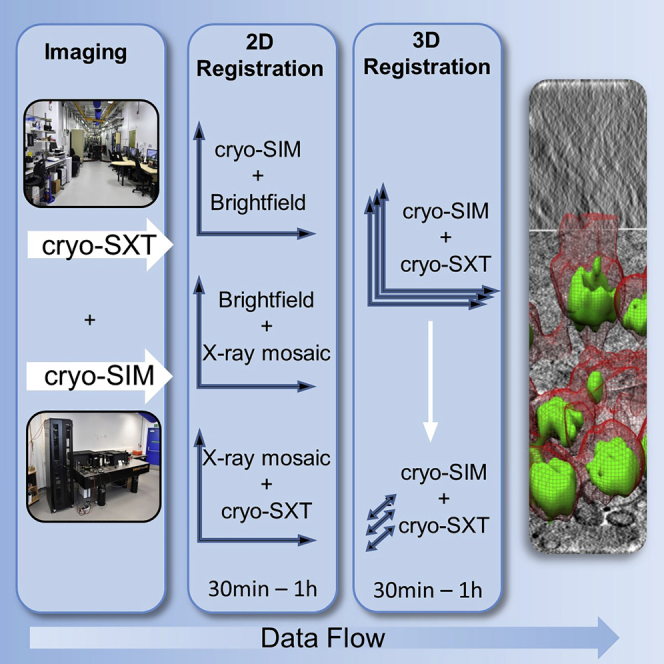

## Before you begin

**Figure 1:**
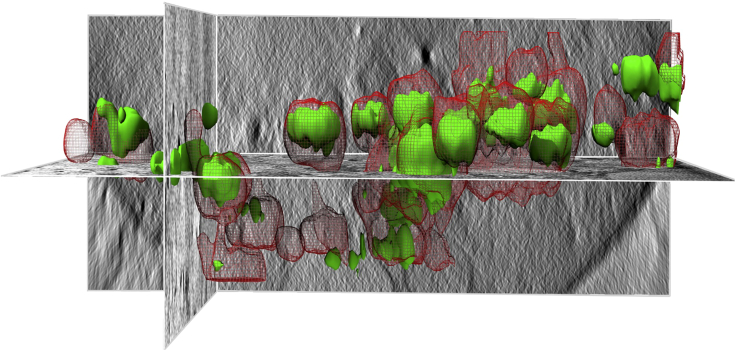
Illustration of correlative imaging of an area of a cell combining cryo-SIM and cryo-SXT. Image data from reovirus infected U2OS cells as described in Kounatidis et al. (2020) The grayscale orthoslices show representative X-ray-generated contrast of cellular vesicles (part of the nucleus can be seen on the bottom right with endosomes clearly delineated in the surrounding cytoplasm). Green volumes are representations of Alexa-488 green fluorescent reovirus localization within endosomes identified by endovesicular concentrations of mCherry-tagged red-fluorescent galectin-3.

Advances in imaging technology are constantly enabling breakthroughs in biological sub-cellular research. Cryo-imaging, specifically, allows for nanometer resolution at near-physiological conditions (Henderson et al., 1990) and correlative cryo-imaging integrates further information about cellular processes. A recent development in correlative cryo-imaging involves a platform developed at the correlative cryo-imaging beamline B24 at the UK synchrotron facility which combines 3D super resolution fluorescence microscopy (cryo-SIM) (Phillips et al., 2020) and soft X-ray tomography (cryo-SXT) (Kounatidis et al., 2020) (Figure 1).

A crucial step in the analysis of imaging data generated across microscopes within an imaging platform is the accurate image co-registration without systematic positional errors or data distortion in 3D. Here we demonstrate the use of the open source Icy (De Chaumont et al., 2012) plugin eC-CLEM (Paul-Gilloteaux et al., 2017) for the registration of SIM and SXT data. eC-CLEM was developed originally for correlating light and electron microscopy images and we present here protocols that allow its adoption for the registration of light microscopy and X-ray tomography volumes in an easy to follow step-by-step process.

### General housekeeping

**Timing: 30 min–3 h**

1. Identify the data and metadata required.

***Note:*** Because SXT and SIM data are collected at different times and on different equipment, it is advisable to have all files in one directory so that you can easily start the registration process. The image files needed are given in Table 1.

2. Ensure you have the necessary processing capacity for 3D data registration.

**Table 1:** Data sets a user is likely to have available for 3D SIM-SXT data registration (required files are denoted with ∗)

Image Type	What is it?	On which instrument was it collected?	Software used	Image data properties				
				File extension	Type	Data type	Pixel size	FOV Dimensions (pixels)
Grid map^a^	Visible light mosaic of wider grid surfaces	cryoSIM	Cockpit	.mrc	mrc	unsigned 16-bit integer	0.125μm	Variable
Brightfield∗	White light bright-field z-stack	cryoSIM	Cockpit	.dv∗	mrc	unsigned 16-bit integer	0.125μm	512×512
SIM	Raw SIM stack	cryoSIM	Cockpit	.dv∗	mrc	unsigned 16-bit integer	62.5nm 125 nm axial	1024×1024
SIR∗	Reconstructed SIM data	cryoSIM	Softworx	.dv∗	mrc	32-bit float	62.5 nm lateral 125 nm axial	1024×1006
Grid map (TXM)	Visible light mosaic of the whole grid	cryoTXM	XRM controller/ Data Explorer (Zeiss)	.xrm/.tif	ole/tiff	unsigned 16-bit integer	0.37 μm	Variable
VLM BF	White light image	cryoTXM	XRM controller/ Data Explorer (Zeiss)	.xrm/tif	ole/tiff	unsigned 16-bit integer	0.37 μm	324×324
VLM FL	Fluorescence image	cryoTXM	XRM controller/ Data Explorer (Zeiss)	.xrm/tif	ole/tiff	unsigned 16-bit integer	0.37 μm	324×324
X-ray mosaic	2D X-ray mosaic of projections including the area of the FOV	cryoTXM	XRM controller/ Data Explorer (Zeiss)	.xrm/.tif	ole/tiff	unsigned 16-bit integer	10 nm or 16nm	Variable
X-ray mosaic annotated∗	2D X-ray mosaic with tomogram locations	cryoTXM	XRM controller/ Data Explorer (Zeiss)	.xrm/.tif∗	ole/tiff	unsigned 8-bit integer (RGB)	10 nm or 16nm	Variable
Tilt series	Raw data	cryoTXM	XRM controller/ Data Explorer (Zeiss)	.txrm/.tiff/.mrc	ole/tiff/mrc	unsigned 16-bit integer	10 nm or 16nm	946×946
Tilt series (referenced)	Raw data (referenced)	cryoTXM	XRM controller/ Data Explorer (Zeiss)	.txrm/.tiff/.mrc	ole/tiff/mrc	unsigned 16-bit integer	10 nm or 16nm	946×946
Tomogram/s of area of interest∗	3D stack of images	cryoTXM	IMOD or similar	.rec∗	mrc	Variable	10 nm or 16nm	1000×1000 or 1600×1600

a The grid map is generated using StitchM, developed at Diamond Light Source, available at: https://github.com/DiamondLightSource/StitchM.

A generic 64-bit personal PC or laptop will suffice but due to the size of imaging data files (circa 1 GB for each 3D volume) and complex 3D processing required, sufficient RAM and hard disk allocation will ensure expediency. An ideal configuration would include at least 16 GB of RAM (preferably 32 GB) with several GB of free storage space for paging.

3. Ensure you have the necessary software in place:
  a. If fluorescence data of the same field of view in different channels collected consecutively appear misaligned along the z axis , this is likely to be due to chromatic shift (Matsuda et al., 2018). If chromatic shift correction is needed, download Chromagnon (Matsuda et al., 2018) from https://github.com/macronucleus/Chromagnon and place at your preferred directory. The version used in this protocol is Chromagnon v0.85.
  b. Download Icy from http://icy.bioimageanalysis.org/download/ and install according to site instructions. The version used in this protocol is Icy 2.1.0.1.
  c. Configure Icy to make use of the maximum available RAM: memory settings are automatically set up at the first run of Icy, depending on your system and the memory available. You can change these using the *preferences* panel (*Max Memory* in *General* panel of *Preferences*)
    **CRITICAL:** You will also need Java installed for Icy to work. Download the correct version for your operating system at the link given in the Icy FAQ section: http://icy.bioimageanalysis.org/faq/. Attention should be paid to the Java version needed (1.8 is advised and 64-bits is mandatory; use the provided links on the Icy website)
    ***Note:*** Launch Icy: in the output Panel, you should see the correct version of the Java machine installed (e.g., for Java Oracle *Java(TM) SE Runtime Environment 1.8.0_231-b11 (64 bit)*and the message *VTK 6.3.0 library successfully loaded.* If this latter does not appear, check the Icy FAQ and in specific https://forum.image.sc/t/vtk-not-loading-in-icy-version-2-1-x/47248/2 (permanent link).
  d. Download the ec-CLEM plugin. The version used in this protocol is 2.0.1.
    i. ec-CLEM should be installed from Icy directly http://icy.bioimageanalysis.org/plugin/ec-clem/. This protocol uses the developmental version of the plugin, not in the public Icy plugin repository at the time of writing; the full release is expected within 2021. To install the developmental version before its public release, download the file at: https://github.com/anrcrocoval/ec-clem/raw/master/binary/ec_clem-2.0.1-SNAPSHOT.jar and copy it in <your-icy-directory>/plugins/perrine/easyclemv0/ (The path is the same for all operating systems). It will then appear as plugin *easyCLEM* the next time Icy is run. Once the public release of this version is released, replace the developmental version with ec-CLEM version 2.0.1 (download direct from the Icy *online plugin* tab). This will thereafter appear as ec-clem when Icy is run. Installed versions of Icy plugins appear in the application windows and can be verified in the Icy Changelog at http://icy.bioimageanalysis.org/plugin/ec-clem/#changelog.
  e. Download the *Correlative View* plugin if you want to create overlay images with no resolution loss at the end of 3D alignment.
    i. Follow the instructions at https://github.com/anrcrocoval/CorrelativeView/. This protocol uses the developmental version of the plugin, not released under the Icy plugin repository at the time of writing (release scheduled for early 2021). To test it, download the .jar from: https://github.com/anrcrocoval/CorrelativeView/raw/master/binary/correlativeview/CorrelativeView.jar and copy it in ‘<your-Icy-directory>/plugins/perrine/correlativeview/’.

***Note:*** It is advisable that you have an additional visualization package such as Fiji (Schindelin *et al.*, 2012) (ImageJ) installed locally to allow fast and independent data and metadata display when needed. Fiji can be downloaded at https://imagej.net/Fiji/Downloads.

4. Become familiar with the generic use of the software that will be employed in this process.
  a. Chromagnon – see the documentation at https://github.com/macronucleus/Chromagnon and in the article by Matsuda et al. (Matsuda et al., 2020).
  b. Icy/eC-CLEM – video tutorials are available at http://icy.bioimageanalysis.org/plugin/eC-CLEM/.
  c. Fiji/ImageJ – tutorials are available at https://imagej.nih.gov/ij/docs/examples/index.html.

## Key resources table

**Table:** 

REAGENT or RESOURCE	SOURCE	IDENTIFIER
**Deposited data**		

U2OS- reovirus 2 h after infection (Area 1)	EMPIAR-10416	BioImage Archive: S-BIAD19

**Software and algorithms**		

Fiji	Schindelin et al. (2012) ‘Fiji: an open-source platform for biological-image analysis’, *Nature methods*, 9(7), pp. 676–682.	Fiji/Downloads - ImageJ
Icy	De Chaumont et al. (2012) ‘Icy: An open bioimage informatics platform for extended reproducible research’, *Nature Methods*, pp. 690–696. https://doi.org/10.1038/nmeth.2075.	Home - Icy – Open Source Image Processing Software (bioimageanalysis.org)
Chromagnon	Matsuda et al. (2018) ‘Accurate and fiducial-marker-free correction for three-dimensional chromatic shift in biological fluorescence microscopy’, *Scientific reports*, 8(1), pp. 1–14.	https://github.com/macronucleus/Chromagnon
eC-CLEM	Paul-Gilloteaux et al. (2017) ‘eC-CLEM: flexible multidimensional registration software for correlative microscopies’, *Nature methods*, 14(2), pp. 102–103.	http://icy.bioimageanalysis.org/plugin/eC-CLEM

**Other**		

Cryo-structured illumination microscope (Diamond Light Source, Beamline B24)	Phillips et al., 2020. CryoSIM: super-resolution 3D structured illumination cryogenic fluorescence microscopy for correlated ultrastructural imaging. *Optica*, *7*(7), pp.802-812.	n/a
Cryo-transmission X-ray microscope (Diamond Light Source, Beamline B24)	Zeiss	n/a

## Step-by-step method details

### Chromatic shift correction

**Timing: 5 min–15 min**

The channels in reconstructed SIM data files (SIR) may be misaligned in x, y, and z with respect to each other due to: (a) mechanical misalignment in the optical path and/or (b) chromatic shift between wavelengths. Both can be corrected using the software Chromagnon (Matsuda et al., 2018). Here are two methods for SIM channel correction depending on the data:

#### Method 1:

This method will only work if there are common fluorescent features in both channels. No reference image is required, only the image to be aligned.

1. Add the SIR data (for 2-channel data files) to both the reference and target fields in Chromagnon. Alternatively, if each fluorescence channel is available in separate files add one as the ‘target’ and one as the ‘source’.
2. Press ‘Run all’.
3. Evaluate the result visually (automatic display by Chromagnon); fluorescence signal should align well in all dimensions.

#### Method 2:

This method uses a reference matrix supplied by the user facility that provides microscope access (it will be specific to the particular optical setup in place at the time of data collection). It can be used when there are no common features in the fluorescence signal in different channels. The reference file is a ‘chromagnon.csv’ file which contains the alignment parameters for the system and has been obtained beforehand from calibration images. It can be used to batch-process multiple images at once.

4. Obtain the reference files from the microscope facility/beamline support scientists.
5. Choose the appropriate reference file which matches the laser wavelength and filter used for imaging your sample and add it to the ‘reference’ field.
6. Add the SIR data to be aligned in the ‘source’ field.
7. Check the suffix to be used in the target panel and choose .dv as output format. The output will be a *<filename>_ALN.dv* file.
8. Press ‘Run all’.
9. Evaluate the result visually; fluorescence signal should align well.
10. For batch-processing place all SIR images to be aligned in the source field, and the reference file in the reference field and press ‘Run all’.

***Note:*** When using a single 2-channel file as both reference and target data, Chromagnon will automatically define the lower wavelength channel as the source.

***Note:*** The 3D transformation file *<filename>.dms.chromagnon.csv*, created automatically by Chromagnon, and the log file *.log* should be saved as records.

The output image of this step is the corrected SIR for chromatic shift and should be created with the name *<original_SIR_Filename>_ALN.tif*.

### Stage shift correction

**Timing: 15 min–30 min**

In some cases, particularly if there is a time lag between acquisitions of successive datasets, there may also be shifts in the microscope stage that need to be accounted for. For example, this can occur if a sample has multiple emitters at different wavelengths to be recorded (the cryoSIM microscope can only record two channels per acquisition). In that case, data are recorded in sets of two channels and each time at least one wavelength is re-collected to act as a reference point against the added channels collected.

**Figure 2:**
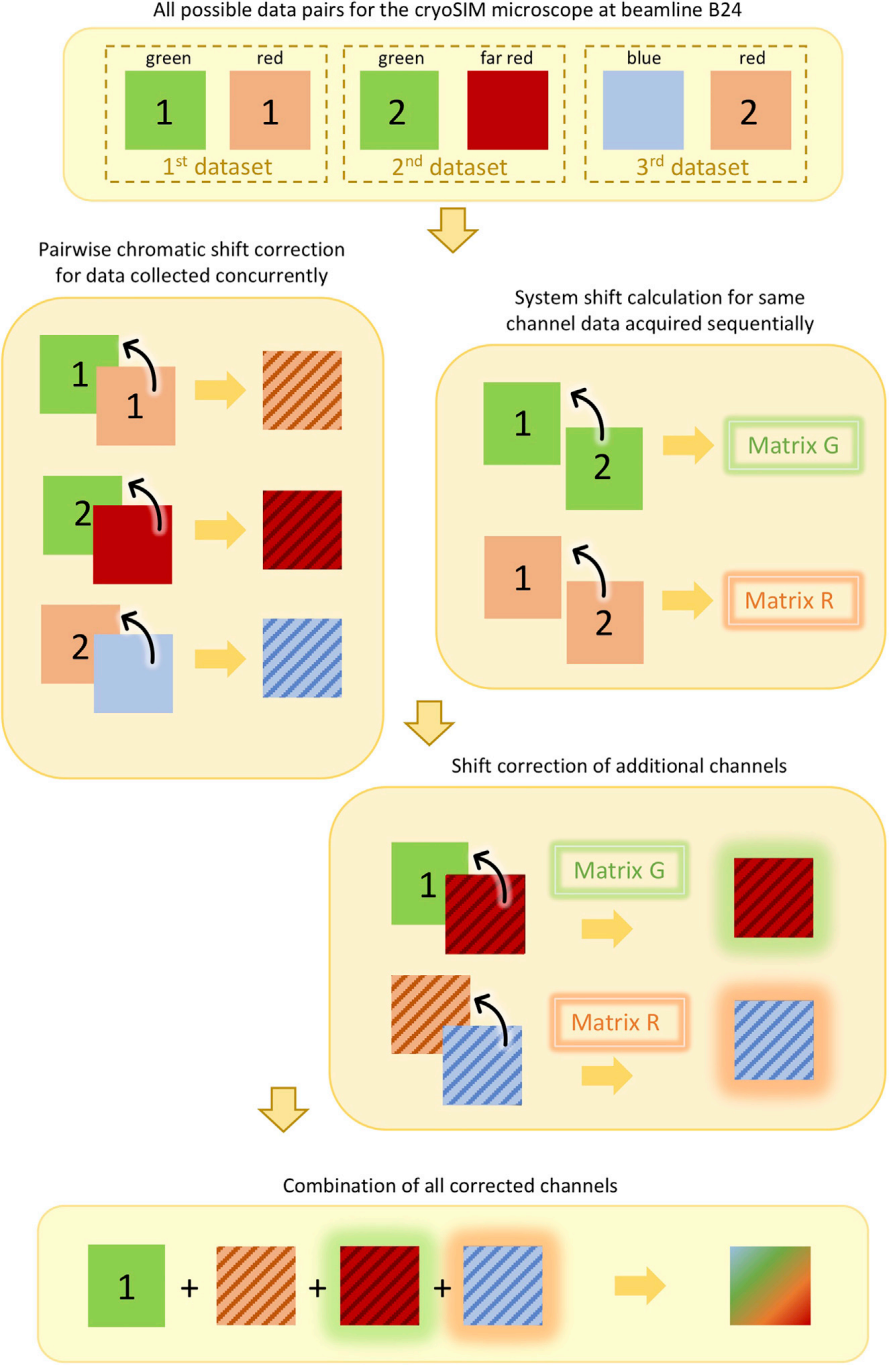
Schematic illustrating the steps in image registration for correcting for chromatic and stage shift for representative 4-channel SIR data Data pairs should be chosen with a view of enabling the calculation of stage drift between successive data acquisition runs. First, the chromatic shift between fluorescence channels is corrected and then the stage-induced drift is calculated from the same channel taken at different time points. The stage drift transformation matrix is then applied to the chromatically corrected channels and finally all channels are merged to obtain a cumulative internally aligned dataset with all the available channels.

See Figure 2 for a schematic of the processing steps.

11. Acquire pairs of images, with one common channel in both pairs:
  a. Designate one image from one channel to be the reference to which all other images will be transformed to. Always add this image in the ‘reference’ part of Chromagnon whenever it is used. In Figure 2 this is the green channel 1, from the first image pair.
12. Perform the chromatic shift correction on each channel:
  a. Follow the same steps as in the **Chromatic Shift Correction** section of this paper.
13. Calculate the stage shift and apply the matrix to correct for stage shift to the other channel(s):
  a. Open Icy.
  b. Open the datasets of the same channel taken at different times. These will contain the same information but will not be entirely co-incident in 3D due to shifts in the microscope setup that can occur between successive data collection cycles.
  c. Open ec-CLEM.
  d. Assign the images as ‘target’ (the reference image) and ‘source’ (the image that you want to transform).
  e. Follow steps d-h from the **Image Registration** section of this paper to co-register the images.
  f. A transformation schema file will be saved in the source image folder.
  g. Open the data corresponding to the second channel from ‘source’ <filename>._SIR_ALN.dv.
  h. In ec-CLEM, go to ‘Advanced Usage’→ ‘Apply transformation matrix’.
  i. Choose the channel to transform and the transformation schema file that was just created and click the play button to apply the transform.
  j. Save the transformed data.
  k. Repeat the steps if there are other images that require alignment for stage shift.
14. Merge all final aligned channels into one image using Icy (Sequence→Merge) or ImageJ/Fiji.

### Pre-processing of images for registration

**Timing: 5 min–30 min**

SIR and SXT 3D image stacks are converted to 2D z axis maximum/minimum intensity projections for ease of processing during the first 2D registration step. This can be done in either Icy or Fiji/ImageJ.

#### Pre-processing in Icy

**Figure 3:**
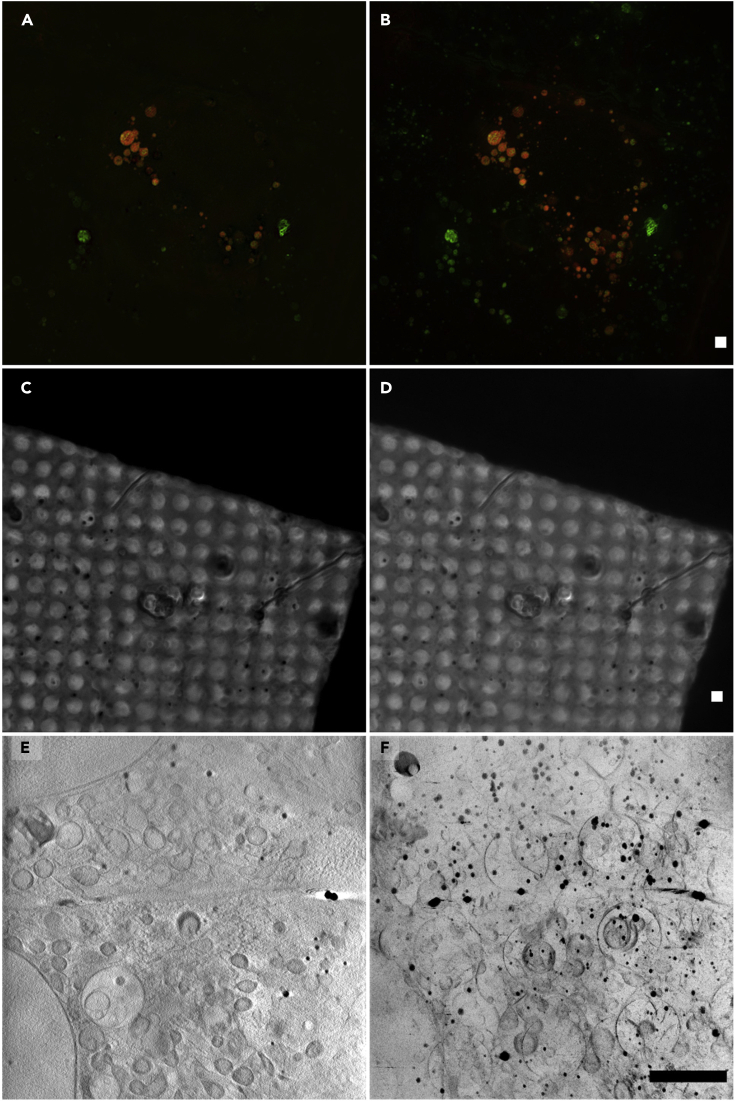
Slices from data z-stacks compared to their contrast-adjusted 2D maximum/minimum intensity projections The projection images demonstrate the highlighted information for registration. (A and B) (A) Slice from an SIR image stack and its total data maximum intensity projection (B). (C and D) (C) Slice from a bright-field z-stack taken on the cryoSIM and its total data minimum intensity projection (D). (E and F) (E) Slice from an X-ray tomogram and the corresponding minimum intensity projection of all data slices (F). The scale bars are 2 μm.
  c. Choose minimum as the projection type for the bright-field image stack and the X-ray tomogram stacks (Figures 3C–3F).
  d. Manually adjust the contrast using the minimum and maximum sliders on the histogram in the bar on the right in Icy to obtain the best contrast for viewing features in the image.
  e. Save these projection images to use in the next steps.

15. Open Icy and search for one of the relevant plugins: Projection or Intensity Projection.
  a. Choose to project along the z axis.
  b. Choose maximum as the projection type for the SIR image stack (Figures 3A and 3B).

#### Pre-processing in Fiji

16. Open Fiji and go to Image→ Stacks → Z Project:
  a. For the SIR stack, choose ‘maximum intensity’ for the projection type (Figures 3A and 3B).
  b. For the bright-field image stack and the X-ray tomogram stacks, choose ‘minimum intensity’ for the projection type (Figures 3C–3F).
  c. Manually adjust the contrast if needed by going to Image→Adjust→Brightness/Contrast.
  d. Name and save these projection images, which will be used in subsequent registration steps.

***Note:*** Before applying the projections, it may help to remove out-of-focus slices from the start and/or end of a stack (for example caused by the sample being tilted in the z direction), to obtain a clearer final projection image (use Image → Stacks→Tools→Make Substack)

***Note:*** It is possible to reduce the file size of the reconstructed X-ray tomogram stacks in the same way by deleting slices which do not contain any features (commonly at the beginning and end of the stack).

### Image registration

**Timing: 30 min–1 day depending on data size**

There are three main steps in Correlative Light X-ray Tomography (CLXT) data registration. First, three separate 2D registrations are done. Then a new transformation schema is created and applied to the 3D image stacks. Finally, the 3D stacks are registered in z.

***Note:*** Steps one and two can be done on any standard computer but the 3D registration in step 3 is more computationally intensive and requires a system with enhanced RAM capacities (at least 16 Gb).

***Note:*** Any files generated by ec-CLEM will be saved in the same folder as the input source image.

17. Compute the 2D image registrations:
  a. Ensure the following images are saved in the same directory:
    i. 2D SIR (image obtained from Z-projection of maximum intensity, fluorescence microscopy).
    ii. 2D brightfield (image, minimum intensity z-projection).
    iii. 2D X-ray (mosaic image, typically annotated with the regions of interest during data acquisition).
    iv. 2D X-ray tomogram (minimum intensity z-projection from 3D X-ray tomograms).
  b. Open Icy and an image pair from each row shown in Table 2.
    **CRITICAL:** For each image opened in Icy, check that the metadata, such as pixel size and image dimensions, are correct. The metadata are shown in the right-hand bar of the Icy main display. Inspect the metadata and edit if needed (occasionally metadata are captured or read incorrectly by software and ec-CLEM will not be able to compute the transformation correctly if these details are incorrect).
    ***Note:*** Records of all metadata are saved in <filename>.xml files in the working directory (generated automatically when a file is first opened in Icy). Deleting an .xml file will allow Icy to read the metadata anew next time the corresponding dataset is read if needed.

**Table 2:** Pairs of images required in each of the 2D transformations that are to be done first in order to compute the total 2D relocation transformation matrix needed next

Source image	Target image
2D maximum intensity projection of SIR image stack	2D minimum intensity projection of bright-field image stack
2D minimum intensity projection of bright-field image stack	X-ray mosaic
X-ray mosaic	2D minimum intensity projection of X-ray tomogram
  c. Open eC-CLEM and assign the source/target images according to Table 2.
  d. Choose the transformation model to be ‘Rigid’ and the noise model to be ‘Isotropic’.
  e. Press the *play* button.
  f. Add fiducial markers to each of the images, moving paired points to corresponding features in each image. Rotate the field of view of an image if it helps with finding the same area in both images by right-clicking and dragging the mouse cursor. Use image features such as:
    Fiducial markers e.g., gold nanoparticles.
    High contrast cellular features e.g., nuclei or lipid droplets.
    Extraneous features e.g., cracks in ice or foreign objects such as dust particles.
    Features of the grid support film such as holes and tears.
    ***Note:*** If it is difficult to locate corresponding features in each image to place any fiducial markers, refer to the troubleshooting section in this paper.
    **CRITICAL:** When finding locations for placing fiducial markers, only rotate the image using the right mouse button as this will not permanently alter image properties. Note that images may be flipped with respect to each other. Do not use Icy’s flip tool or other image transformation tools that are not a part of ec-CLEM, since such changes will not be tracked by ec-CLEM and the final transformation schema will not be correct. Instead, place 2–3 points and *compute the transform* in ec-CLEM which will reposition the image during the transformation, after which you can place more points and re-compute the transform. See more information on this in the troubleshooting section.
    ***Note:*** In Icy, you can zoom in and out of an image using the mouse scroll wheel. The image will zoom to the location of the mouse pointer on the image.
  g. Click on *update transform* after placing 3–10 points. The transformation files will be automatically saved in the same folder as the source images. Three files are saved per computation: a .csv file containing the transformation matrix, and two .xml files with the suffixes ‘.transformation’ and ‘.transformation_schema’ which contain information on the transformation. The files required for computing the final 3D transformation are the .xml file with the suffix ‘transformation_schema’.
  h. Add more points if required and re-compute the transform. The two images can be locked at this point by choosing the same numbered lock icon in the top left of both views to make it easier to synchronously zoom into both images at the same location.
    ***Note:*** Lock 1 and Lock 2 will fully synchronize windows. Lock 3 synchronizes in 2D as well as zooming in/out, but not in z. At this stage in the protocol, use Lock 1. Check that the transformation is accurate by merging the images (*merge* button in ec-CLEM). The color and intensity of each image can be adjusted in the histogram bar in Icy located under the Sequence tab.
  i. Click on *stop* and check the output panel. It should say that the transformation has been saved and state its directory:
    Transformation schema saved at:
    *<filepath>\<filename>.transformation_schema.xml*
    CSV format transformation saved at:
    *<filepath>\<filename>.transformation.csv*
    XML format transformation saved at:
    *<filepath>\<filename>.transformation.xml*
    You do not need to save the registered image for this protocol. All the information required for subsequent steps is located within the xml files.
  j. Repeat with the remaining data combinations from Table2 until you have three transformation schema files, one for each data pair.

18. Create a final transformation schema and apply to the 3D image stacks:
  a. Open ec-CLEM in Icy, click advanced options and choose compute cascading transformation schema.
    i. Add the transformation schema files (with suffix *.transformation_schema.xml*) for each image pair in the same order as shown in Table 2.
    ii. Add a *Save* location for the new 3D transformation schema file, ensuring you add *.xml* at the end of the filename you choose.
    iii. Press play to compute and save it.
  b. Open the 3D image stacks corresponding to the aligned 2D SIR image (<filename>_SIR.dv or <filename>_SIR_ALN.dv if chromatic correction was performed) and the X-ray tomogram in Icy.
    i. In the ec-CLEM advanced options, click *Apply Transformation Schema*
    ii. Apply the new 3D schema to the SIR image stack.
    iii. Save the transformed image stack (*Save As…*).

19. Register the 3D stacks in the z direction:
  a. Open the transformed SIR image stack and the 3D X-ray tomogram image stack (.rec) in Icy and use ec-CLEM in the same way as detailed in Step 17f to add fiducial markers, but this time to register images in 3D.
  b. Note that you should keep the RIGID option to ensure no data deformation.
    ***Note:*** Be aware that choosing fiducials that are highly concentrated in one area of the volume examined can result in 3D alignments that are skewed. If RIGID 3D alignment fails consistently to produce full volume alignment (some areas are better aligned than others) and the fiducials chosen are spread evenly across data volumes, AFFINE alignment can be chosen on the understanding that the SIM data will suffer a degree of deformation likely along the z axis.
    ***Note:*** Lock both volumes (they are now pre-aligned in 2D) using Lock 3. Note that the datasets are not aligned in z at this stage of the process and common features should be present at similar x and y but different ‘heights’ in z.
  c. Place between 5–10 points or until any further improvement cannot be seen, using features such as nanoparticles as your fiducials.
  d. Click *stop.* All process parameters are saved within *.csv* and *.transformation_schema* files.
  e. Save the transformed image stacks and/or the merged images. This step can be time consuming and you can consider the ‘SaveAsTifFast plugin’ (simply search for it in the Icy search bar). This uses a faster ImageJ version of saving.

20. The fully aligned datasets can now be viewed in any relevant 3D visualization package such as ChimeraX (Goddard et al., 2018; Pettersen et al., 2021).

**CRITICAL:** Ensure you use ‘Lock 3’ when doing z channel registration in ec-CLEM. Each dataset needs to be at the corresponding z slice that features a shared feature before a fiducial marker is placed there (shared features will be located at different depths in the respective stacks before alignment). To retrospectively change the position of a fiducial in z, either switch to 3D VTK view or use the ROI panel, select an ROI and change its Z position.

***Optional:*** Z-alignment can also be done by observing the SIR image stack as an orthogonal view. In Icy click on the drop-down arrow in the top left of the image and change it from 2D to OrthoViewer. This may help to more accurately place the point in the center of the point spread function of fluorescent points but active rendering is computationally demanding and is therefore likely to slow hardware performance.

### Evaluation of results

**Timing: 5 min–30 min**

**Figure 4:**
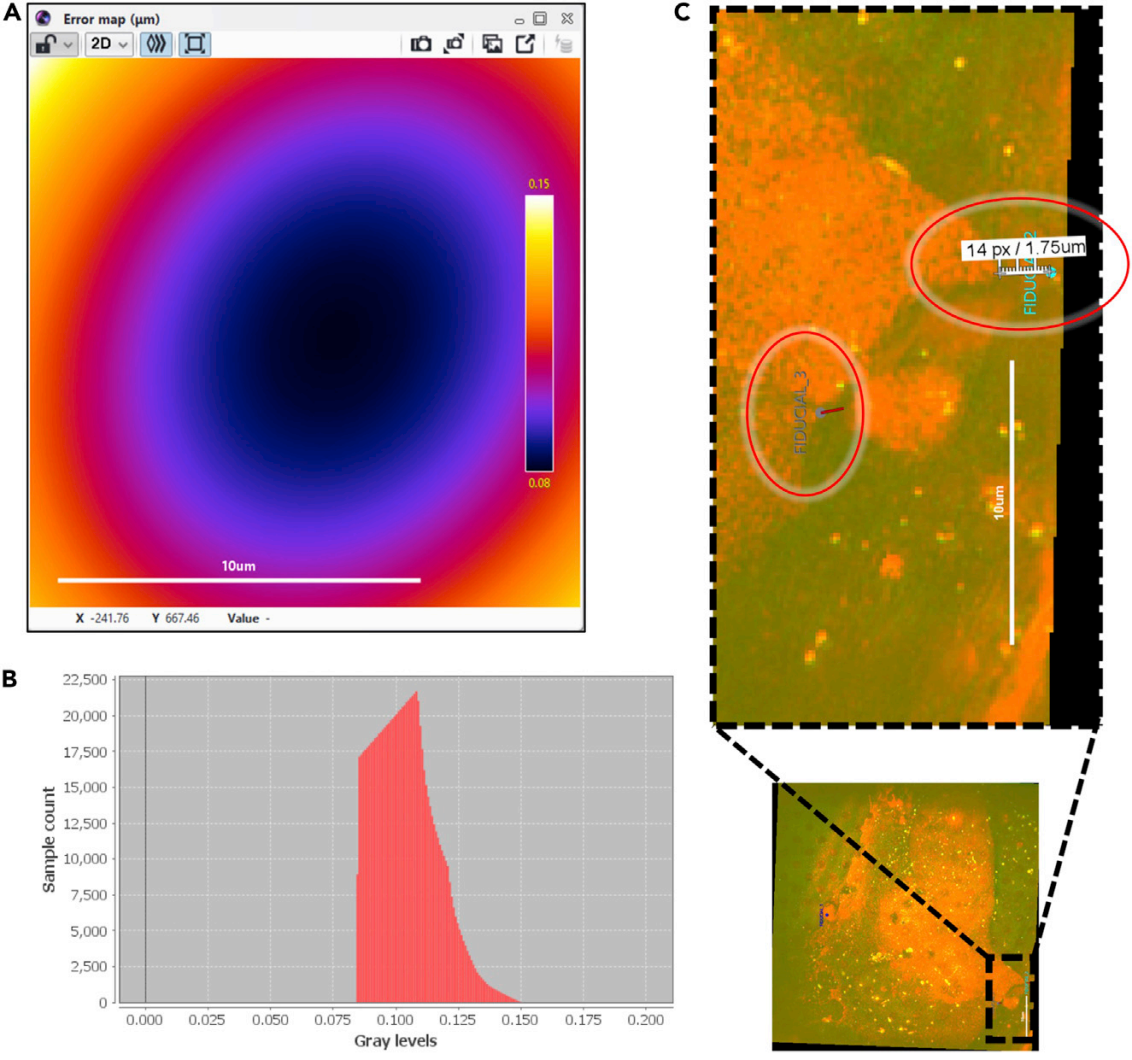
Demonstrations of the two types of registration error calculation in ec-CLEM (A) Error map. (B) Corresponding histogram to measure the distribution of the registration error. (C) Error between individual points, shown as a red line near the fiducial markers.

Accuracy of the co-registration can be evaluated in ec-CLEM, through two types of error calculations: an error map showing the general distribution of positional offsets in the whole image (Figure 4A), or individual fiducial marker errors (Figure 4C). The histogram of the error map can be generated in Icy to view the distribution of the error (Figure 4B)

21. Generate an error map in ec-CLEM:
  a. After computing a transform, click *Compute the whole predicted error map.*
  b. The error map can be scaled by adjusting the histogram.
  c. The appearance of the scale bar or color bar can be toggled under ‘layer’ in the right bar shown in Icy.
  d. The maximum and minimum values on the color bar show the values of the maximum and minimum error.
  e. Install the histogram plugin in Icy (search for “histogram” in Icy) to plot the error map distribution. The width of the plot on the x axis corresponds to the registration error and the y axis is the number of points with each error value. Also see Figure S3 in Kounatidis et al., 2020 for more details of measuring the registration error.
22. Measure the error between points used as landmarks by clicking on ‘show measured error’:
  a. Zoom in to the fiducial points to see red lines corresponding to error bars.
  b. Use the ‘ruler helper’ tool in Icy to measure the length of each red line to obtain error magnitudes.

### Creating an image overlay of the tomogram on the SIR image

**Timing: 5 min–30 min**

Once the transformation schema has been computed, it can also be used to overlay the tomogram on the full-size SIR image, without any loss in resolution, using the *Correlative View* plugin in Icy.

**Figure 5:**
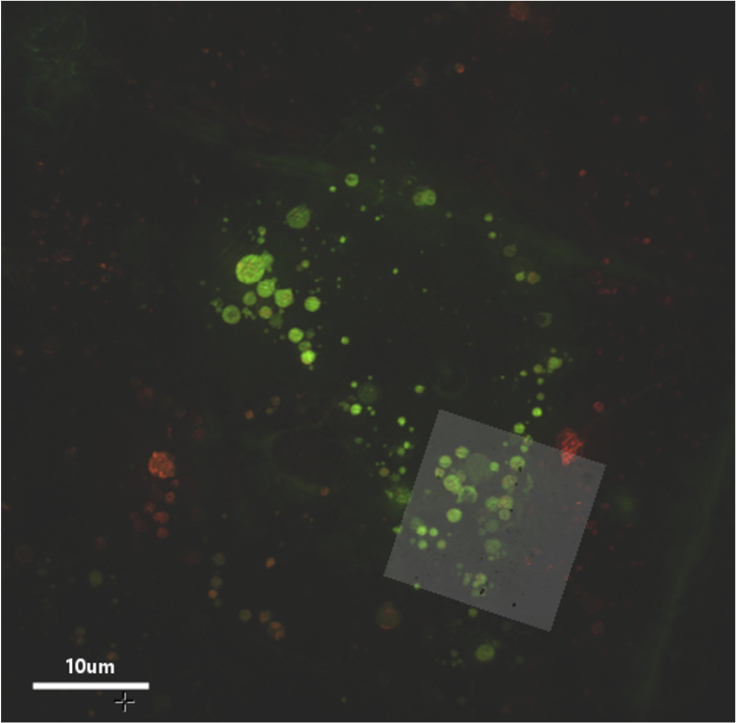
Overlay image produced of the tomogram transformed onto the SIR volume using the Correlative View Plugin This can be done by inverting the transformation matrix used to transform the SIR image onto the tomogram.

23. Download the *Correlative View* plugin and save it in your Icy plugins folder.
24. Invert the transformation schema:
  a. In ec-CLEM, go to *Advanced*→*Invert schema.* Choose the final combined transformation schema you created in step 18.
  b. Ensure that the schema has the file extension .xml (manually rename if needed).
  c. Create a name for the new file, adding .xml at the end.
  d. Click *run.* Two new .xml output files will be created, the file you will need in the next step should have the suffix ‘matrixtransfo’.
25. Open the maximum intensity SIR image in Icy:
  e. Click the drop-down icon menu in the top-left of the image in Icy and find the *Correlative View* plugin.
  f. Choose the minimum intensity tomogram image that you want to superimpose.
  g. Choose the inverted transformation file that ends in ‘matrixtransfo’.
  h. The overlay image will appear without any resolution loss (Figure 5).

## Expected outcomes

Co-registered images after alignment in ec-CLEM are shown in Figure 2. The accuracy of registration required depends on the number and size of the features that can be tracked and used as fiducial markers. In general, for CLXT data, a successful image registration should show an average error of under 100 nm for a well-defined feature.

**Figure 6:**
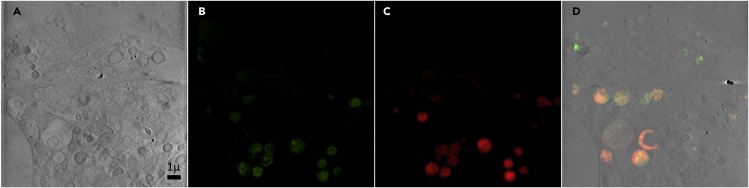
Transformed images after correlation and their overlay (A) Slice from an X-ray tomogram. (B and C) Transformed SIR data channels after registration in x, y, and z using ec-CLEM. (D) Overlay showing co-localization of the fluorophores inside vesicles.

The final overlay of a representative X-ray tomogram and the corresponding cryoSIM data are shown in Figure 6.

## Limitations

Given imaging data that has been collected and processed with care, this protocol should work for any CLXT experiment. However, poor data quality, commonly resulting from poor sample preparation, may cause difficulties in locating features for fiducial markers. Adverse factors include imaging artefacts, ice crystals, insufficient feature intensity, poor contrast, and few or poorly distributed correlation markers. Such issues can largely be avoided by following various published guidelines for obtaining optimum CLXT data (Demmerle et al., 2017; Harkiolaki et al., 2018; Wu et al., 2020; Okolo et al., 2021; Vyas et al., 2021). In this protocol we have used rigid transformations to avoid any image warping. Should the sample have suffered deformation during handling or data collection it may be necessary to use an affine transform (with shearing) or a spline transformation (with warping) to align imaging data.

## Troubleshooting

### Problem 1

Difficulty in locating the same areas in different images

In step 17f it may be difficult to find obvious sample features for placing marker points for registration in ec-CLEM, especially for the bright-field and X-ray mosaic image pair because these may be rotated or flipped due to sample positioning in the different microscopes.

### Potential solution

Coarse and Fine registration

Coarse registration is done to first align the images so they both have the same orientation. This then makes it easier to spot further features which can be used as fiducials in fine alignment.

You can first look for large sample features which can be easily distinguished such as:

• Cell nuclei.
• Junctions e.g., where two cells meet each other.
• Non-biological features on the EM grid surface which are close to large objects, such as nuclei, so can be easily identified.
• Groups of particles which form shapes, such as a line or a triangle, which can be distinguished in both datasets.
• Cracks in the vitreous ice that surrounds the cells.

**Figure 7:**
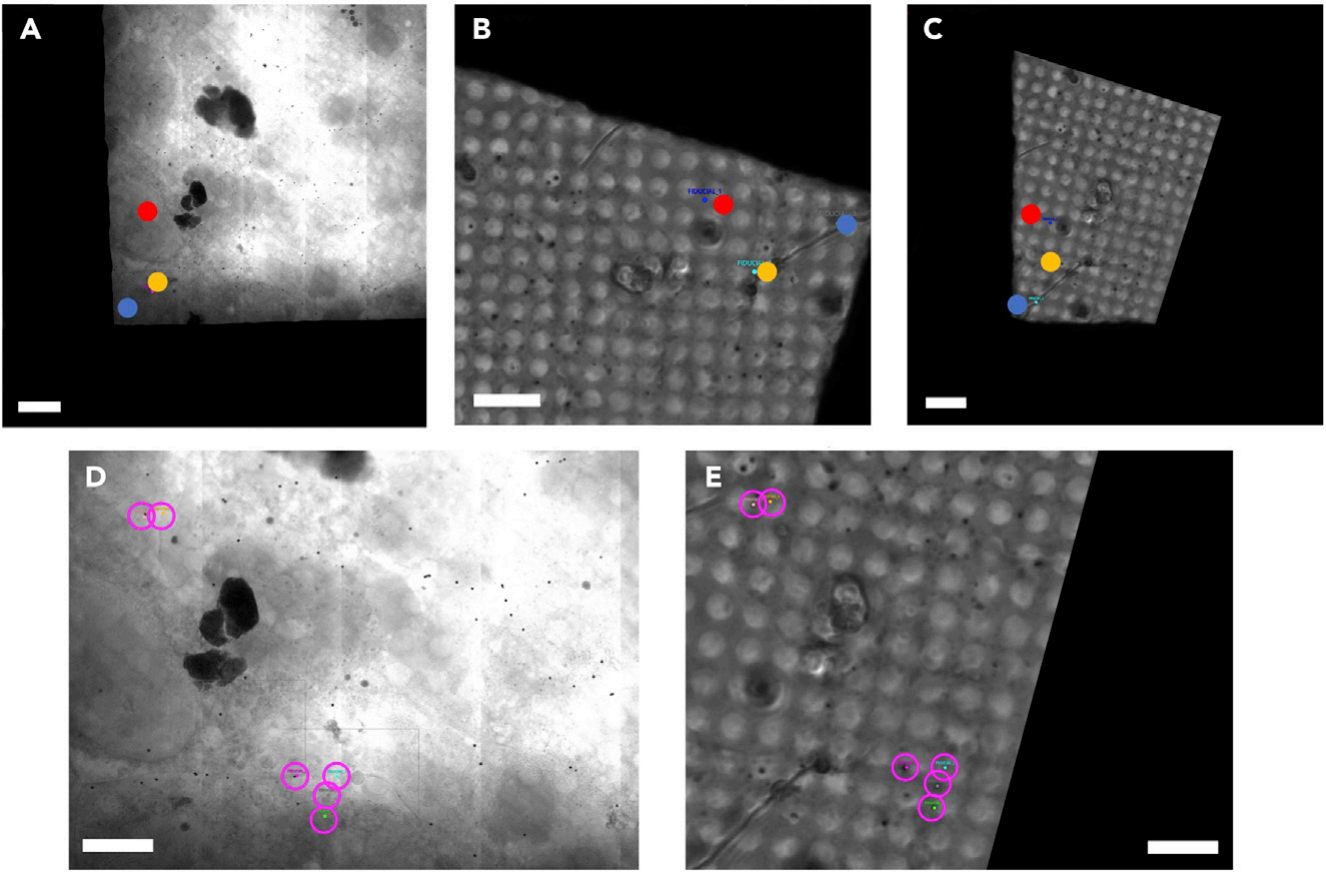
Example of using 2-step coarse and fine registration This method can be useful for images where it is difficult to initially locate the same areas. (A) X-ray mosaic image. (B) Bright-field image. (C) Transformed image after coarse registration using just a few low accuracy points. (D and E), close-up images after coarse registration, where more fiducial markers have been placed. The magenta outline shows the new fiducial markers such as on lipid droplets or nanoparticles which have now been placed accurately. The scale bars are 10 μm.

Perform a two-step coarse and fine registration in ec-CLEM (Figure 7).

In ec-CLEM, place three marker points approximately in locations which correspond to the features such as at the center of a cell nucleus. (Figures 7A and 7B)
Update the transformation. The images should now be in the same orientation. (Figure 7C)
Lock the two images and zoom in to find more features.
Place more accurate fiducials on smaller features such as nanoparticles/lipid droplets or similar. (Figures 7D and 7E)
Delete the first 3 initial fiducial markers in each image or reposition them to more accurate locations.
Update the transformation to obtain the accurate transformation schema file.

### Problem 2

Image transformation is incorrect

In steps 1g and 3c the image may transform incorrectly for several reasons. For example, if it is a stack it may show the same image in each slice, or if it is a 2D image it may disappear after updating the transformation in ec-CLEM.

### Potential solution

Check the metadata

This is usually due to incorrect pixel size settings. Re-open the image and check the metadata in Icy again, including the z pixel size if transforming an image stack. Ensure the dimensions are correct.

The final 3D transformation may be incorrect if the 2D transformations have a large registration error. Check the transformation accuracy of the 2D images beforehand by merging the reference and target images after each 2D registration.

If there are still issues with the registration, use the sample dataset in this study (See the data and code availability section) to practice the registration steps.

## Resource availability

### Lead contact

Further information and requests for resources and reagents should be directed to and will be fulfilled by the corresponding authors, Dr. Maria Harkiolaki (lead contact) (maria.harkiolaki@diamond.ac.uk) and Dr. Perrine Paul-Gilloteaux (technical contact) (perrine.paul-gilloteaux@univ-nantes.fr).

### Material availability

This study did not generate new unique reagents.

### Data and code availability

Original imaging data referenced in the manuscript is deposited at the BioImage Archive (https://www.ebi.ac.uk/biostudies/BioImages) and EMPIAR (https://www.ebi.ac.uk/pdbe/emdb/empiar/). The accession numbers for the data are EMPIAR: EMPIAR-10416 and BioImage Archive: S-BIAD19.
